# The Mechanism of HDAC2 Inhibitors on Chronic Pancreatitis Pain

**DOI:** 10.1055/a-2561-8065

**Published:** 2025-05-07

**Authors:** Xiang-tian Zeng, Wen-hui Chen, Ding-wen Zhong, Qi-xin Dai, Yong He, Rong-Qiang Ye, Xiu-lin Xiao, Yong-hui Liao

**Affiliations:** 1 Central Supply Service Department, Ganzhou People's Hospital, Ganzhou, Jiangxi, People's Republic of China; 2 Department of Hepatopancreatobiliary Surgery, Ganzhou People's Hospital, Ganzhou, Jiangxi, People's Republic of China

**Keywords:** chronic pancreatitis, spinal nerve, HDAC2, ERK-Sp1 pathway, pain

## Abstract

**Background:**

Chronic pancreatitis (CP) is marked by persistent inflammation and fibrosis of the pancreas, often causing severe abdominal pain. The pain mechanism involves complex interactions between pancreatic inflammation and spinal nerve activity. Histone deacetylase 2 (HDAC2) is implicated in neural processes and pain modulation, making it a potential target for CP pain management.

**Aim:**

This study investigates HDAC2's role in CP pain and evaluates the effects of its inhibition in a CP rat model.

**Methods:**

CP was induced in male Sprague–Dawley rats using dibutyltin dichloride (DBTC). HDAC2 expression in spinal and pancreatic tissues was assessed through western blotting, quantitative Real-Time PCR, and enzyme-linked immunosorbent assay (ELISA). Pain sensitivity was evaluated using paw withdrawal tests. Co-cultures of AR42J pancreatic acinar cells and F11 spinal neurons were used to explore pancreatic–neural interactions. Chromatin immunoprecipitation (ChIP) and promoter assays examined HDAC2 transcriptional regulation.

**Results:**

HDAC2 expression was significantly elevated in CP rats, which also displayed increased pain sensitivity and higher inflammatory markers (interleukin [IL]-1β [IL-1β], tumor necrosis factor-α [TNF-α], IL-6, and chemokine ligand 2 [CCL-2]). HDAC2 inhibition reduced pain sensitivity and pancreatitis. Co-culture experiments revealed that pancreatic inflammatory mediators upregulate HDAC2 in neurons. ChIP identified Sp1 as a regulatory factor for HDAC2, with the extracellular signal-regulated kinase-Specific protein 1 (ERK-Sp1) pathway critical for its expression.

**Conclusion:**

HDAC2 is crucial in CP pain sensitization and inflammation. Its inhibition reduces pain and inflammation, offering potential for targeted pain management in CP.

## Introduction

Chronic pancreatitis (CP) is a persistent inflammatory disease of the pancreas characterized by progressive fibrosis and irreversible damage. 1 The condition is often associated with severe abdominal pain, which is a significant clinical symptom and a major cause of reduced quality of life in patients. 2 The pain mechanism in CP is complex and involves multiple pathways, including the sensitization of spinal neurons. 3 4 Recent studies have indicated that chronic inflammation can lead to alterations in spinal cord function, contributing to the persistent pain experienced in CP. 5 6 7 8 The interplay between pancreatic inflammation and spinal nerve activity is crucial for understanding the pain mechanism in CP.

Histone deacetylase 2 (HDAC2) is a key enzyme involved in the regulation of gene expression through chromatin remodeling. 9 10 HDAC2 has been implicated in various neural processes, including memory formation, synaptic plasticity, and neurodegeneration. 11 12 In the context of spinal cord function, HDAC2 plays a critical role in modulating neural activity and pain perception. Studies have shown that HDAC2 expression can influence the excitability of spinal neurons and impact pain pathways, making it a potential target for pain management in chronic inflammatory conditions. 13 14

The involvement of HDAC2 in pancreatitis has garnered research interest due to its role in inflammation and pain modulation. In pancreatic tissue, HDAC2 expression has been observed to increase during inflammatory states. 15 This upregulation is believed to exacerbate pain by enhancing neural sensitization and inflammatory responses. 15 Investigating the role of HDAC2 in CP could provide insights into new therapeutic strategies for managing pancreatitis-associated pain.

This study aims to investigate the role of HDAC2 in the pain mechanism of CP and evaluate the effects of HDAC2 inhibition on pain modulation and inflammation in a CP rat model. We hypothesize that HDAC2 is highly expressed in the spinal cord during pancreatitis and contributes to pain through neural conduction inhibition. The study will use a combination of pharmacological interventions, molecular biology techniques, and behavioral assays to elucidate the underlying mechanisms.

## Materials and Methods

### Animal Model and Treatment

This study used male Wistar rats supplied by Shanghai Slac Laboratory Animal Co., Ltd. (Shanghai, China). The 8-week-old rats were housed at room temperature under a 12-hour light–dark cycle. The rats were divided into two groups: a sham operation group ( *N* = 6) and a CP group ( *N* = 12). CP was induced in the CP group via tail vein injection of dibutyltin dichloride (DBTC; 5 mg/kg; Sigma, Germany). The sham operation group will receive an injection of an ethanol and glycerol mixture to monitor the progression of pancreatitis.

The treatment plan includes the initial administration of DBTC and assessments during the fourth or fifth week postinjection. Additionally, at week 4, CP group was randomly divided into two groups on average; one group received intrathecal administration of HDAC2 inhibitor santacruzamate A (SCA; MedChemExpress, Shanghai, China) will be administered intrathecally once daily, and related experiments will be conducted 1 hour after each injection; the other group did nothing. The study was approved by the Animal Use and Care Committee for Research and Education of Ganzhou People's Hospital (Jiangxi, People's Republic of China; No. TY-DKY2024-016-01) and also the ethical guidelines.

### Behavior Measurements

Pain sensitivity was assessed using paw withdrawal mechanical threshold (PWMT) and paw withdrawal thermal latency (PWTL) tests. 13 16 These assays measure the mechanical and thermal pain thresholds of the rats to evaluate pain sensitivity changes due to CP and HDAC2 inhibition.

### Hematoxylin and Eosin Staining Procedure

The fixed tissue was embedded in paraffin, sectioned into 4-μm thick slices, and then mounted and dried. The sections were sequentially processed through xylene, ethanol, and water baths. Subsequently, they were stained with hematoxylin for 3 to 5 minutes, rinsed in water, differentiated in a differentiation solution, rinsed again in water, treated with bluing reagent, and washed under running water.

Next, the sections were dehydrated in 85% and 95% ethanol for 5 minutes each, followed by staining with eosin for 5 minutes. Finally, the sections were cleared in ethanol and xylene and mounted with neutral resin. Examination and image capture were performed using an Olympus microscope.

### Cell Co-culture

AR42J pancreatic acinar cells and F11 spinal nerve cells were sourced from ATCC. AR42J and F11 cells were cultivated separately using the appropriate culture medium, ensuring both cell types were in the logarithmic growth phase. AR42J cells were seeded in the upper chamber of a transwell insert, with the culture medium containing cholecystokinin (CCK; MedChemExpress, Shanghai, China) to simulate the pancreatitis environment. F11 cells were seeded in the lower chamber of the transwell insert using the same culture medium. the transwell insert was placed into a cell culture plate for co-culture, typically for 24 to 48 hours. the culture medium was collected from both the upper and lower chambers for cytokine analysis.

### Small interfering RNAs (siRNA) Interference

The HDAC2-specific RNAi was custom-designed and synthesized by Shanghai Genechem Co., Ltd. (Shanghai, China). The specific sequence of the HDAC2-targeting RNAi was 5′-CCGTGAAGCTGAACCGTCA-3′, ERK1-targeting RNAi was 5′- CCTGAATTGTATCATCAACAT-3′, Sp1-targeting RNAi was 5′-CCCAAGTTACTGCCTTATGAT-3′. For constructing the lentiviral vector, U6-MCS-Ubi-EGFP served as the backbone structure, while GV118 acted as the vector. Following transfection into 293T cells, a lentivirus package containing either LV-shRNA (carrying the RNA interference sequence) or LV-NC (control sequence) was obtained. The resulting titer reached approximately 1 × 10 ^9^ transducing units/mL.

### Western Blot Analysis

Tissues were homogenized and lysed on ice using a lysis buffer for 30 minutes, followed by disruption using ultrasonic waves for tissue homogenates or cell samples. The mixture was then centrifuged at 12,000 rpm for 15 minutes at 4 °C. Protein concentration was determined using a Bicinchoninic Acid Assay (BCA) protein assay kit. Protein samples were denatured by adding buffer, separated by electrophoresis, and transferred to a polyvinylidene fluoride (PVDF) membrane. The membrane is blocked with 5% non-fat milk for 1 hour, incubated with primary antibodies overnight at 4 °C, and incubated with secondary antibodies for 1 hour at room temperature the next day. Protein bands are visualized using freshly prepared enhanced chemiluminescence (ECL) reagents. The primary antibodies required are anti-actin, anti-ERK1/2, anti-phospho-ERK1/2, anti-Sp1 (Cell Signaling Technology, Danvers, MA), anti-phospho-Sp1 (pThr453; Abcam, Inc., Cambridge, MA), anti-HDAC2, anti-phospho-methyl ethyl ketone (MEK), and anti-MEK (Abcam, Inc., Cambridge, MA).

### Total RNA Extraction and Quantitative Real-Time PCR

Total RNA was extracted from tissues or cells using TRIzol reagent (Invitrogen, Waltham, MA) according to the manufacturer's instructions. cDNA synthesis was performed using the high-capacity cDNA Reverse Transcription Kit (Applied Biosystems, Waltham, MA). Gene-specific primers were obtained from PrimerBank ( http://pga.mgh.harvard.edu ). Quantitative real-time PCR (qRT-PCR) was performed using SYBR Green PCR Master Mix (Takara Bio, Inc., Shiga, Japan) on an ABI 7900HT Fast Real-Time PCR System. The relative expression levels were determined using the 2 ^−ΔΔCT^ method and normalized to the endogenous control gene Glyceraldehyde-3-phosphate dehydrogenase (GAPDH).

### Enzyme-linked Immunosorbent Assay

Enzyme-linked immunosorbent assay (ELISA) was performed to quantify the protein levels of tumor necrosis factor-α (TNF-α), interleukin (IL)-1β (IL-1β), IL-6, and Chemokine Ligand2 (CCL-2) in cells and pancreatic tissues (BioTNT, Shanghai, China). Samples were incubated with specific antibodies, and cytokine concentrations were determined through a colorimetric reaction.

### Plasmid Construction

All PCRs were performed using Pfu Ultra Hotstart DNA polymerase (Agilent) in a PCR thermal cycling apparatus (Thermo fisher). Verification of all constructs was performed by DNA sequencing. To construct the HDAC2 promoter plasmid, we amplified the HDAC2 promoter region (−481 to +141 bp) from genomic DNA with the primers 5′-TGAAACACGTGGGAATATCG-3′ and 5′-GCCAGACAAGAGGGCTGAC-3′. The PCR product was then inserted into the pCRblunt vector (Sangon Biotech, Shanghai, China) and sequenced. Subsequently, the product was re-amplified using the primers 5′-GATctcgagTGAAACACGTGGGAATATCG-3′ and 5′-cttaagcttgccagacaagggctgac-3′, digested with XhoI and *Hind* III, and subcloned into the XhoI and *Hind* III sites of the pGL4.11 [luc2P] vector (Sangon Biotech, Shanghai, China).

### Luciferase Activity Assay

Luciferase activity was measured using a luminometer (TD-20/20; Turner Biosystems) following the manufacturer's instructions with the Dual-Luciferase Reporter Assay System (Sangon Biotech, Shanghai, China). HEK293 cells were seeded at a density of 1 × 10 ^5^ cells per well in six-well plates and cultured for 24 hours before transfection with the relevant plasmids. Cells were transfected with 1.0 μg of pGL4.11 [luc2P] plasmid (Sangon Biotech, Shanghai, China) containing the HDAC2 promoter region (−481 to +141 bp), along with pcDNA3.1-FLAG-Sp1 plasmid at varying amounts (1.0, 2.0, and 4.0 μg). After 24 hours of incubation posttransfection, luciferase activity was measured using the TD-20/20 luminometer and the Dual-Luciferase Reporter Assay System, following the manufacturer's protocol. Transfection efficiency was normalized using the co-expressed pGL4.75 [hRluc/CMV] plasmid (Sangon Biotech, Shanghai, China) at 0.02 μg per well.

### Chromatin Immunoprecipitation

Chromatin immunoprecipitation (ChIP) was performed as previously described. 17 The immunoprecipitated DNA was purified, resuspended in water, and subjected to PCR. To amplify the HDAC2 promoter region, the following primers were used: 5′-TGAAACACGTGGGAATATCG-3′ and 5′-GCCAGACAAGAGGGCTGAC-3′. PCR products were separated by electrophoresis on a 1.5% agarose gel containing ethidium bromide and visualized under UV illumination.

### Statistical Analysis

SPSS 22.0 was used for statistical analysis. Statistical tests for data analysis included a two-tailed Student's *t* -test, paired *t* -test, and Fisher's exact test for comparison. Data were presented as means ± SD. *p* -values less than 0.05 were considered statistically significant.

## Results

### Histone deacetylase 2 Expression in Pancreatitis

**Fig. 1:**
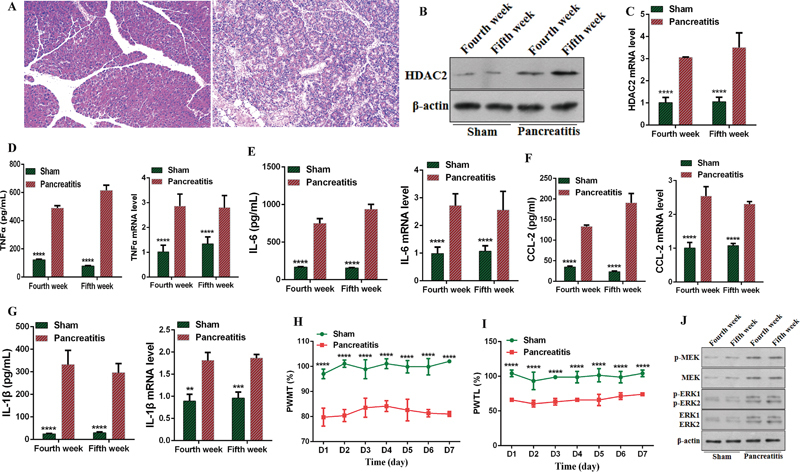
Elevated expression of HDAC2 in the spinal cord of DBTC-induced pancreatitis rats. ( **A** ) H&E staining of rat pancreas. ( **B, C** ) WB and qPCR analysis of HDAC2 mRNA and protein levels in the spinal cord. ( **D–G** ) qPCR and ELISA analyses of TNF-α, IL-1β, IL-6, and CCL-2 expression. ( **H, I** ) Changes in mechanical and thermal hyperalgesia in pancreatitis-induced rats. ( **J** ) WB analysis of differential expression of p-MEK, MEK, p-ERK1, ERK1, p-ERK2, and ERK2. CCL-2, chemokine ligand 2; DBTC, dibutyltin dichloride; ELISA, enzyme-linked immunosorbent assay; HDAC2, histone deacetylase 2; H&E, Hematoxylin and Eosin; IL, interleukin; TNF-α, tumor necrosis factor-α; WB, Western blot. Note: **** means **** *p* < 0.0001.

Hematoxylin and Eosin (H&E) staining confirmed the successful induction of CP in DBTC-treated rats. The H&E staining results demonstrated significant fibrosis and inflammatory cell infiltration in the pancreatic tissues of DBTC-treated rats at the fourth and fifth weeks ( Fig. 1A ). Western blot (WB) and qPCR analyses indicated that the expression of HDAC2 in the spinal cord of rats in the CP group was significantly elevated ( Fig. 1B, C ). Furthermore, qPCR and ELISA assays revealed increased levels of the inflammatory factors IL-1β, TNF-α, IL6, and CCL-2 in the pancreatic tissues ( Fig. 1D–G ). These findings suggest the successful establishment of the CP model in rats.

Subsequently, we assessed pain sensitivity in the rats at the fourth and fifth weeks. The results of PWMT and PWTL tests showed that mechanical hyperalgesia and thermal hyperalgesia were significantly increased in the CP group, indicating enhanced sensitivity to mechanical and thermal pain in DBTC-induced CP rats ( Fig. 1H–I ).

Therefore, what signaling pathway mediates the effect of pancreatitis on spinal nerve sensation? Research indicates that ERK/MEK is an upstream regulator of HDAC2, which plays a crucial role in neuropathic pain. Our WB analysis demonstrated that the levels of p-MEK, p-ERK1, and p-ERK2 were significantly elevated in the spinal cords of rats in the CP group ( Fig. 1J ), suggesting that the ERK/MEK signaling pathway was activated in the spinal cord following DBTC-induced CP.

### Correlation between Histone Deacetylase 2 and Pain Behavior

**Fig. 2:**
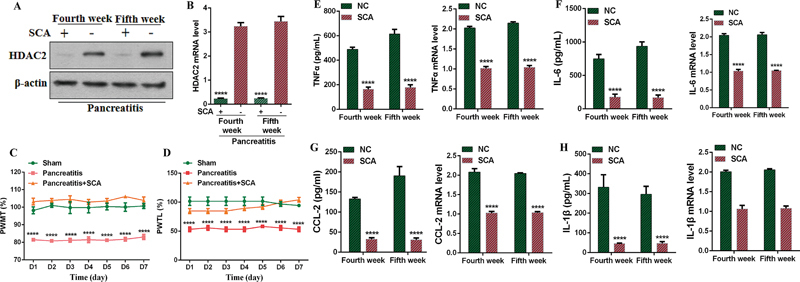
Impact of HDAC2 on pain behaviors in chronic pancreatitis. ( **A, B** ) WB and qPCR analyses of HDAC2 mRNA and protein levels in the spinal cord. ( **C, D** ) Effects of HDAC inhibitor on mechanical and thermal hyperalgesia in rats. ( **E–H** ) qPCR and ELISA analyses of TNF-α, IL-1β, IL-6, and CCL-2 expression. CCL-2, chemokine ligand 2; ELISA, enzyme-linked immunosorbent assay; HDAC2, histone deacetylase 2; IL, interleukin; PWMT, paw withdrawal mechanical threshold; PWTL, paw withdrawal thermal latency; SCA, santacruzamate A; TNF-α, tumor necrosis factor-α; WB, Western blot. Note: **** means **** *p* < 0.0001.

The previous results indicated that rats exhibited significant symptoms of CP at the fourth and fifth weeks following DBTC treatment. To further investigate the role of HDAC2, we administered the HDAC2 inhibitor SCA via spinal catheter injection at the fourth week, observing changes in HDAC2 expression and pain sensitivity over the following 7 days. Our qPCR and WB results confirmed that spinal HDAC2 expression was significantly inhibited by the seventh day ( Fig. 2A, B ). PWMT and PWTL tests conducted over the 7 days demonstrated a reduction in mechanical and thermal hyperalgesia in rats compared with those not injected with SCA ( Fig. 2C, D ). Additionally, we examined changes in the pancreatic tissues of the rats. ELISA results also showed reduced levels of IL-1β ( Fig. 2E ), TNF-α ( Fig. 2F ), IL-6 ( Fig. 2G ), and CCL-2 ( Fig. 2H ) in the pancreatic tissues at this time point. Thus, we speculate that the HDAC2 inhibitor may indirectly alleviate pancreatic inflammation in rats by improving molecular changes in the spinal cord.

### Cellular Mechanisms of Histone Deacetylase 2 Regulation

**Fig. 3:**
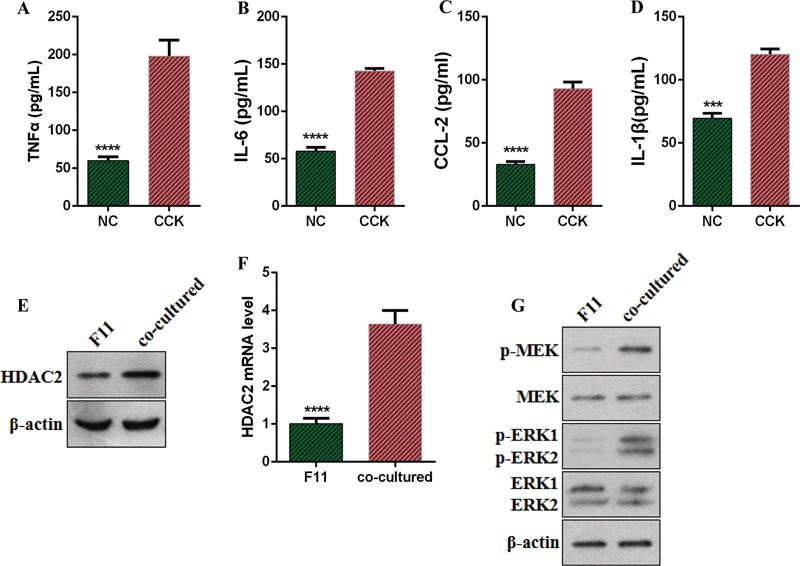
Pancreatic cells promote HDAC2 expression in neuronal cells. ( **A–D** ) CCK treatment-induced changes in TNF-α, IL-1β, IL-6, and CCL-2 in AR42J cells. ( **E, F** ) WB and qPCR analysis of HDAC2 mRNA and protein levels in co-cultured cells. ( **G** ) WB analysis of changes in p-MEK, MEK, p-ERK1, ERK1, p-ERK2, and ERK2. CCK, cholecystokinin; CCL-2, chemokine ligand 2; HDAC2, interleukin; IL, TNF-α, tumor necrosis factor-α; WB, Western blot. Note: *** or **** means *** *p* < 0.001 or **** *p* < 0.0001.

To further explore the relationship between HDAC2 and pancreatic inflammation in the spinal cord, we plan to use the cell co-culture technique for verification. Co-culturing AR42J and F11 cells and stimulating AR42J cells with CCK, ELISA results showed increased levels of IL-1β, TNF-α, IL-6, and CCL-2 in AR42J cells ( Fig. 3A–D ). In F11 cells co-cultured with AR42J + CCK, both mRNA and protein expression levels of HDAC2 also increased ( Fig. 3E, F ). On the other hand, WB results indicated that after co-culturing with AR42J + CCK, p-MEK, p-ERK1, and p-ERK2 levels in F11 cells also increased ( Fig. 3G ). This further suggests that pancreatic inflammatory cells may affect the expression of neuronal cell HDAC2 through certain molecular and intercellular relationships.

### ERK-Sp1 Pathway and Histone Deacetylase 2 Expression

**Fig. 4:**
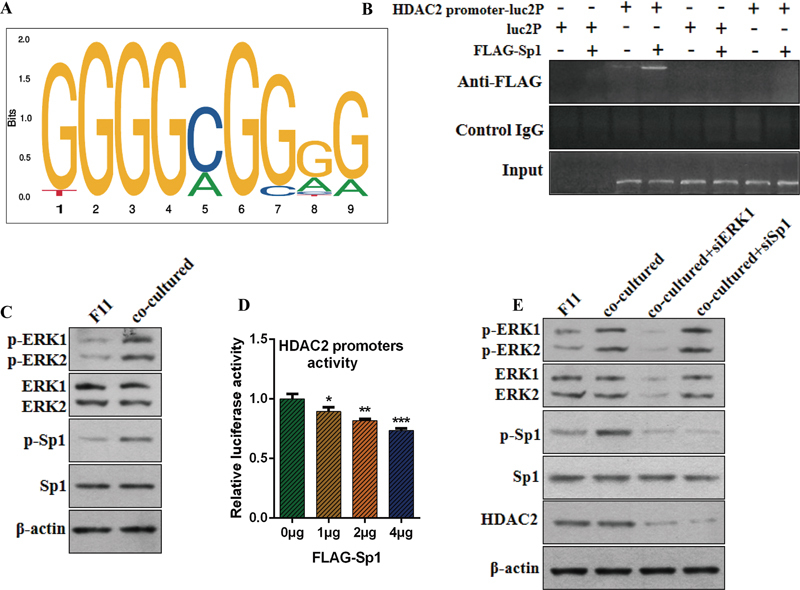
**(A)** Bioinformatics analysis indicated that Sp1 can bind to the promoter region of HDAC2. **(B)** ChIP assay showing Sp1 binding to the HDAC2 promoter region. **(C)** WB analysis showing changes in total and phosphorylated ERK and Sp1 proteins after cell co-culture. **(D)** Analysis of HDAC2 promoter activity. **(E)** Changes in HDAC2 protein levels after siERK1 and siSp1 treatments. * *p* < 0.05, ** *p* < 0.01, or *** *p* < 0.001.

The ERK pathway can influence the expression of HDAC2. Thus, when we interfered with ERK gene expression using siRNA, the protein levels of HDAC2 also decreased correspondingly ( Fig. 4D ), even under the co-culture conditions of F11 with AR42J + CCK. Bioinformatics analysis indicated that Sp1 can bind to the promoter region of HDAC2 ( Fig. 4A ). By constructing vectors containing Sp1 expression sequences and HDAC2 promoter sequences, ChIP experiments demonstrated that Sp1 can bind to the HDAC2 promoter ( Fig. 4B ). On the other hand, WB analysis showed that the levels of p-MEK, p-ERK1, p-ERK2, and p-Sp1 were significantly elevated in F11 cells co-cultured with AR42J + CCK ( Fig. 4C ). Promoter activity assays indicated a significant decrease following treatment with mithramycin A ( Fig. 4D ). After siRNA interference of ERK1 and Sp1, the expression levels of ERK1 and Sp1 significantly decreased, leading to a reduction in HDAC2 expression as well ( Fig. 4E ). Therefore, we conclude that the ERK pathway influences HDAC2 levels by affecting Sp1 phosphorylation.

## Discussion

This study demonstrates that the high expression of HDAC2 in CP is associated with increased pain sensitivity and enhanced inflammatory responses. HDAC2's role in the spinal cord promotes pain sensitization through neural conduction inhibition mechanisms. HDAC2 inhibitors significantly reduce pain and inflammation, indicating their potential in CP pain management. The study finds that HDAC2 expression is regulated by various inflammatory mediators secreted by pancreatic cells, including TNF-α, IL-1β, IL-6, and CCL-2. Co-culture experiments further confirm the interaction between pancreatic cells and spinal neurons, suggesting that inflammation induced by pancreatitis affects not only the local site but also the central nervous system through neuroimmune interaction mechanisms. Inflammatory mediators can affect spinal neurons through intercellular or tissue-specific pathways, leading to increased neuronal excitability and heightened pain perception. 18 19 These findings reveal the crucial role of inflammation in pain mechanisms, providing a theoretical basis for developing therapeutic strategies targeting HDAC2.

The ERK-Sp1 signaling pathway plays a significant role in studies of the nervous system and inflammation. 20 ERK is part of the MAPK signaling pathway, which is critical in cell proliferation, differentiation, and survival. 21 22 Sp1 is a transcription factor that regulates the expression of various genes by binding to specific GC box sequences in DNA. 23 24 ERK phosphorylates Sp1, enhancing its transcriptional activity and affecting the expression of many genes related to the nervous system and inflammation. 25

In the nervous system, the ERK-Sp1 signaling pathway is essential for neuronal survival, neurite outgrowth, and neural plasticity. 26 Previous research has shown that ERK activation can promote neuronal survival and regeneration, while Sp1 is involved in the transcriptional regulation of neuron-specific genes. 20 26 For example, the ERK-Sp1 pathway plays a crucial role in nerve growth factor-induced neurite outgrowth, which is vital for recovery after nerve injury. 27

In inflammatory responses, the ERK-Sp1 signaling pathway also plays a significant role. ERK activation is typically associated with the release of inflammatory factors such as TNF-α and IL-1β. 28 These inflammatory factors can further activate ERK, enhancing the transcriptional activity Sp1, leading to the production of more inflammatory mediators, and forming an amplifying feedback loop. 29 30 HDAC2 is a deacetylase that regulates gene transcription by modulating histone acetylation states. 31 The ERK-Sp1 pathway upregulates HDAC2 expression, contributing to the regulation of inflammatory responses.

In chronic inflammatory diseases like CP and neuropathic pain, abnormal activation of the ERK-Sp1 signaling pathway is closely related to high HDAC2 expression. Inhibiting ERK or Sp1 can effectively reduce HDAC2 expression, thereby alleviating inflammatory responses and pain perception. This finding provides a new target for treating chronic inflammatory diseases. However, this study utilized only the DBTC-induced CP model to evaluate the efficacy of HDAC2 inhibition, which may present model-specific limitations. For instance, potential interactions between DBTC and HDAC2 in neuronal cells cannot be ruled out. Future studies should incorporate alternative CP models, such as caerulein-induced pancreatitis, to further validate the generalizability and robustness of these findings. Additionally, although this study demonstrated that HDAC2 inhibition significantly reduces pain sensitivity and inflammation, further research is needed to investigate the dose-dependent effects and long-term efficacy of HDAC2 inhibitors. Such studies would provide deeper insights into the mechanisms underlying HDAC2's role in chronic inflammatory pain and offer more comprehensive evidence for developing HDAC2-targeted therapeutic strategies. Future research could further explore the application of HDAC2 inhibitors in other chronic inflammatory diseases (such as inflammatory bowel disease) and develop specific therapeutic strategies targeting HDAC2 to improve patients' pain and quality of life.

## Conclusion

This study sheds light on the critical role of HDAC2 in the pain mechanism of CP. The findings support the therapeutic potential of HDAC2 inhibitors in alleviating CP-associated pain and provide a foundation for future research into targeted pain management strategies.
